# Narrow-Leafed Lupin Main Allergen β-Conglutin (Lup an 1) Detection and Quantification Assessment in Natural and Processed Foods

**DOI:** 10.3390/foods8100513

**Published:** 2019-10-18

**Authors:** Elena Lima-Cabello, Juan D. Alché, Jose C. Jimenez-Lopez

**Affiliations:** 1 Department of Biochemistry, Cell & Molecular Biology of Plants, Estacion Experimental del Zaidin, Spanish National Research Council (CSIC), Profesor Albareda 1, E-18008 Granada, Spain; elena.lima@eez.csic.es (E.L.-C.); juandedios.alche@eez.csic.es (J.D.A.); 2 The UWA Institute of Agriculture and School of Agriculture and Environment, The University of Western Australia, Crawley, WA 6019, Australia

**Keywords:** vicilin, 7S-globulins, food allergens, Lup an 1, sweet lupin species, food labelling, processed food

## Abstract

The increasing prevalence of lupin allergy as a consequence to the functional characteristics of a growing number of sweet lupin-derived foods consumption makes the imperious necessity to develop analytical tools for the detection of allergen proteins in foodstuffs. The current study developed a new highly specific, sensitive and accurate ELISA method to detect, identify and quantify the lupin main allergen β-conglutin (Lup an 1) protein in natural and processed food. The implementation of accurate standards made with recombinant conglutin β1, and an anti-Lup an 1 antibody made from a synthetic peptide commonly shared among β-conglutin isoforms from sweet lupin species was able to detect up to 8.1250 ± 0.1701 ng (0.0406 ± 0.0009 ppm) of Lup an 1. This identified even lupin traces present in food samples which might elicit allergic reactions in sensitized consumers, such as β-conglutin proteins detection and quantification in processed (roasted, fermented, boiled, cooked, pickled, toasted, pasteurized) food, while avoiding cross-reactivity (false positive) with other legumes as peanut, chickpea, lentils, faba bean, and cereals. This study demonstrated that this new ELISA method constitutes a highly sensitive and reliable molecular tool able to detect, identify and quantify Lup an 1. This contributes to a more efficient management of allergens by the food industry, the regulatory agencies and clinicians, thus helping to keep the health safety of the consumers.

## 1. Introduction

Lupine is a legume that belongs to the genus *Lupinus* and is included in the *Leguminosae* family, which is of great interest to the food industry, similarly for chickpeas, beans, peanuts, soya bean, lentils, and peas. *Lupinus* comprises between 200–600 different species [1]. However, only the four known as the sweet lupin group have gained interest since they are used in human food production [2] for their low levels of alkaloids [3] contained in their seeds. These four species include *Lupinus albus* (white lupine), *Lupinus angustifolius* (blue lupin or narrow-leafed lupin, NLL), *Lupinus luteus* (yellow lupine) [4], and *Lupinus mutabilis* (pearl or Andean lupin) that are mostly cultivated and consumed in central and South America [5].

Current interest for lupin seeds as a new functional food is growing [6], and the seeds from the sweet lupin species are becoming a crucial and alternative source of proteins for human consumption with nutritional and nutraceutical properties [7,8,9,10]. Sweet lupin species are a promising source of innovative ingredients for functional food, particularly those from the vicilin or β-conglutin family, which are the most abundant proteins in NLL seeds [11]. Among the most frequently found, flour is one of the basic products and a common form to use lupin seeds as an ingredient in a wide range of food i.e., bread, cake, pasta, pizza, sausage, spices, cream cheese, tofu, jam [12]. In addition, lupin might often replace soya bean, egg white, and milk in vegan food [5,13], and is used as a functional ingredient in gluten-free food [12,14].

In the last five years and due to this increased nutraceutical knowledge, the number and range of commercially available lupin based products increased. In this regard, the rising lupin-derived products consumption, and the growing number of allergy reactions in sensitized persons have also increased. The routes of sensitization not only arise as primary sensitization to ingested lupin seed proteins, but also occur due to the cross-reactivity in atopic subjects sensitized to seed proteins from other legumes, particularly to soybean and peanut [15,16,17]. A third route appears to be the occupational allergy in people that works daily with lupin flour and lupin derived products [18].

Although the prevalence in the general population of lupin allergy is still unknown [19], it has been estimated to be in the range of 0.3% to 8%, and particularly in children being 5% [5,13,20]. It is important to identify the lupin seed proteins involved in allergy reactions [21,22], with storage proteins being the main lupin allergens [23], particularly from the vicilin family (β-conglutins), which were named as Lup an 1 in NLL (WHO/IUIS Allergen Nomenclature Subcommittee). Due to the significant increase of reported cases of lupin allergy, and in order to keep safety among the population, the seeds from this legume together with soya bean and peanut were included in the European Union regulations (Regulation (EU) No. 1169/2011) as foods prone to induce allergy reactions. There is also a mandatory declaration in the labelling of the pre-packaged food ingredients, and overall, it provides information to consumers of these potential sources of food allergens. Thus, this demands very accurate and sensitive, highly specific quantitative methods to identify the main allergen protein content in food in order to identify the cross-contamination that justifies the precautionary labelling because of the presence of lupin as an ingredient. Thus, the aim of the present study was to develop a new highly specific ELISA method for the detection, identification and quantification of the lupin main allergen β-conglutin (Lup an 1), and the assessment of this method in natural and processed food. Since lupin flour is being mainly used as an ingredient in bakery products, vegetarian and vegan based products, this analytical method was validated in natural (non-processed) food and processed (fermented, cooked, boiled, roasted) food such as flour, bread, and biscuits; as well as others lupin based alternative foods as spreading, sauce, cheese, pickles, drink, butter, meat. This study also includes data from these commercial foodstuffs differing in their labelling (“may contain lupin traces”, “lupine contained”, “lupine content not included on the food package”).

## 2. Material and Methods

### 2.1. Bioinformatics Analysis of β-Conglutin Protein Sequences

The sequences of seven available isoforms of NLL β-conglutin protein were retrieved from the NCBI database (accession number F5B8V9, F5B8W0–F5B8W5 corresponding to β1 to β7). A multiple sequence alignment and subsequent analysis was performed using ClustalW software (https://www.ebi.ac.uk/Tools/msa/clustalw2), based on Blosum62 matrix (BLOck SUbstitution Matrix) [24], and viewed using the Jalview viewer 2.2 (http://www.jalview.org). The Bioedit v 7.0.5.3 (http://www.mbio.ncsu.edu/BioEdit/bioedit.html) software was used to calculate the sequence identity matrices.

### 2.2. Analysis of the Antigenicity of β-Conglutin Proteins

The prediction of antigenicity for these β-conglutin protein isoforms was performed. Antigenic epitopes were determined on the basis of the following parameters: hydrophobicity [25]; amino acid surface accessibility [26,27]; antigenicity methods such as Hopp and Woods hydropathy [28], Welling [29], Parker [30], BepiPred-2.0 (sequential β-cell epitope predictor, http://www.cbs.dtu.dk/services/BepiPred), and Kolaskar and Tongaonkar [31]. The results were confirmed using the antigenicity prediction of GenScript service (https://www.genscript.com/antigen-design.html).

### 2.3. β-Conglutin Proteins Structure Modelling

The conglutin β1 protein sequence (F5B8V9) was retrieved and used for searching the best structural templates in the Protein Data Bank (PDB) (http://www.rcsb.org). The suitable templates for this sequence were selected using BLAST server (http://ncbi.nlm.nih.gov). To improve the best final templates identification and selection, the software BioInfoBank Metaserver (http://meta.bioinfo.pl) specializing in fold recognition homology was used, as well as the Swiss-model server (swissmodel.expasy.org). The best four identified templates (1uij, 2phl, 3s7e, and 2eaa) were retrieved from the PDB database, and implemented for homology modelling. The conglutin β1 protein model was built by the implementation of SWISS-MODEL via the ExPASy web server (swissmodel.expasy.org) using these top PDB closely related structural templates.

The structural errors in the initial structural 3D models were identified by using ProSA (prosa.services.came.sbg.ac.at/prosa.php), obtaining the first overall quality estimation of each model using QMEAN4 (swissmodel.expasy.org/qmean/cgi/index.cgi). The final structure of β-conglutin proteins was subjected to energy minimization using GROMOS96, which was implemented in DeepView/Swiss-PDBViewer v3.7 (spdbv.vital-it.ch) improving the van der Waals contacts and correcting the stereochemistry. The quality of the final models was assessed by assessing the protein stereology with PROCHECK (www.ebi.ac.uk/thornton-srv/software/PROCHECK) and ProSA programs, as well as the protein energy using ANOLEA (protein.bio.puc.cl/cardex/servers/anolea). The suitability of the model on the basis of the number of protein residues in the favoured regions was assessed by Ramachandran plot statistics. 

To define the potential functional and interacting areas/clusters in the protein, the electrostatic Poisson-Boltzmann (PB) potentials were calculated by using APBS (DeLano Scientific LLC) molecular modelling software implemented in PyMOL 0.99 (www.pymol.org). The electrostatic PB potential values are given in units of kT per unit charge (k Boltzmann’s constant; T temperature).

### 2.4. Construction of the Expression Plasmid

The protein expression in bacteria was achieved using the pET28a(+) vector (Novagen) with some modifications, such as an N-terminal 6xHis Tag. Vector pUC57 was used for cloning a synthetic gene encoding β-conglutin protein (GenBank HQ670409, β1), connected by restriction enzyme linker sequences, NcoI and XhoI (GenScript). The genetic construct included the expression vector pET28a(+)-conglutin β1-6xHis-Tag, which was obtained through the digestion of the pUC57- conglutin β1 construct with NcoI and XhoI restriction enzymes, followed by ligation of the β1 fragment into the pET28a(+) vector.

### 2.5. Overexpression of Conglutin β1

The final genetic construct containing the expression vector with the conglutin β1 gene was transformed into Rosetta™ 2(DE3) pLysS Singles™ Competent Cells (Novagen) for β1 expression. The protein expression was accomplished by using an auto-induction method [32]. Briefly, a colony of *Escherichia coli* containing the construct was isolated and grown for 24 h in ZY-medium plus kanamycin (50 μg/mL) at 37 °C in constant shaking (190 rpm). The culture was diluted 1:175 in Studier medium to grow for 7 h until the cell density reached 0.6 OD at 600 nm. The overexpression induction was achieved by adjusting the temperature to 20 °C during 17 h. The bacterial cell pellet was collected by centrifugation at 5000× *g* at 4 °C. The bacterial pellet was washed three times with PBS, pH 7.5, and after removing the supernatant, the pellet cell was flash frozen using liquid nitrogen. The resulting pellet was stored at −80 °C until further use.

### 2.6. Purification of the Recombinant Conglutin β1 Protein

Overall, β1 purification was performed following the company’s recommendations (Qiagen) for Histidine tagged proteins. Briefly, the main steps comprised the cells breaking, followed by affinity chromatography using nickel-NTA spin columns, and linking a 6xHis-Tag at the C-terminal end of the β1 protein. The elution of this protein from the column was performed with an increasing imidazole concentration gradient (25–350 mM), collecting 2 mL fractions. All the fractions containing the protein were analyzed using SDS-PAGE, and those containing a single band with the expected molecular weight were pooled and dialyzed against PBS during 2 days with buffer changes every 12 h. The resulting samples were then aliquoted and flash frozen in liquid nitrogen to be kept at −80 °C until further uses. The purity of the protein samples was >95%. The typical yields were ~25–55 mg/mL.

### 2.7. Antibody Production Against β-Conglutin

The performance and specificity of an antibody highly depends on the nature of the binding to its protein target. A polyclonal epitope-specific antibody was developed using a combination of a lineal epitope identification and characterization (antigenicity assessment) in the target protein sequence and an affinity capture approach involving synthesized peptides. 

The sequences currently available of seven NLL β-conglutin genes were retrieved from UNIPROT database (https://www.uniprot.org). The alignment of these sequences was analyzed to find a commonly shared antigenic peptide among the seven NLL β-conglutin proteins variants following an analysis of antigenicity described in previous sections, finding the following sequence: Nt– VDEGEGNYELVGIR –Ct. A synthetic peptide with this sequence was produced (Agrisera, Vännäs, Sweden). The chosen sequence (up to 14 amino acids + terminal cysteine) was synthesized in a 15 mg immunograde purity scale without any further modifications. In order to elevate the antigenicity of the synthetic peptide, 2 mg of it was coupled to a carrier protein, KLH, which is the keyhole limpet hemocyanin copper-containing protein, with a molecular size >450 kDa.

The KLH coupled synthetic peptide was used for animal (rabbits) immunization. The typical amount of antigen for a standard immunization protocol is ~500 µg/animal, and a preferable concentration >1 mg/mL dissolved in PBS. Five animals were subjected to a program of three immunization rounds (105 days long) with the same antigen. After 80 days, the test samples were delivered to our laboratory for evaluation, where sera, after this period of immunization, was tested to check for the capability to detect its target protein and also to show the potential background signal from these different sera in Western blot assays using recombinant and lupin seed protein extracts. The animals’ analyzed polyclonal sera having target-specific antibodies directed towards linear epitope and giving Western blot bands of correct molecular weight against β-conglutin proteins and low or no background was used for further immunization and obtaining the sera. After the third immunization, all serum samples were checked for the presence of antigen specific antibodies using ELISA. A further proof of high specificity and no background produced by the antibody was obtained by a screening of pre immune serum. Testing the pre-immune samples made sure that no background signals were detected in the molecular weight region of the protein under investigation.

Furthermore, the antibody capture and clean up (affinity-purified) from antiserum was performed from the final rabbit immune serum by a liquid chromatography system against the same synthetic peptides as the affinity column tag. This step contributed to obtaining a higher specificity of the antibody, but also reduced the amount of the available antibody in the final serum.

### 2.8. Food and Biological Samples Used for Lupin Allergen Detection and Quantification

The different commercially available foods containing variable quantity of lupin seed were purchased from different supermarkets. More detailed information of each product is described in Table 1.

### 2.9. Proteins Extraction

The total protein (including allergen proteins) extractions were performed from the different food and biological samples. The samples were homogeneously grinded with a polytron homogenizer (Kinematica Polytron™ PT 2500E, VWR), always keeping the samples in a water-ice cool bath. For these proteins, extractions were used as the extraction buffer containing 100 mM Tris, pH 7.4, 250 mM NaCl, 1 mM EGTA, 1 mM EDTA, 1% Triton X-100, 0.5% Sodium deoxycholate, Protease inhibitor cocktail and 1 mM PMSF. The homogenates were centrifuged at 10,000× *g* to remove all the gross material and to keep the supernatant. The high NaCl concentrations contained in the extraction buffer assures specific vicilin proteins extraction [33] as β-conglutin (Lup an 1).

For the protein extraction from tissues, the samples were dissected and kept at a low temperature on ice-water to prevent proteolysis. Then, 50 mL of complete extraction buffer was added to 1 g of the sample in a tube to be homogenized with the polytron homogenizer. Afterward, the blades of the homogenizer were rinsed with the extraction buffer, and the homogenized sample was maintained at a constant agitation for 2 h at 4 °C for the proteins’ final extraction to the media. A centrifugation for 20 min at 15,000× *g* and 4 °C was performed, and the supernatant was placed on ice-water to make the aliquots (containing the soluble protein extract) in a fresh, chilled tube and to store the samples at −80 °C. The lupin drink was analyzed as it was in the bottle. 

### 2.10. Gel Electrophoresis and Western Blots

The analyses of the protein extracts were achieved by mixing each sample individually with 6× protein sample buffer and heated to 95 °C for 5 min. The proteins were separated on commercial 4–20% gradient TGX gels (Bio-Rad, Hercules CA, USA). The molecular weight markers used for stained gels were Precision Plus Protein™ Dual Colour Standards (BioRad). The separated protein bands were visualized in a Gel Doc™ EZ Imager (BioRad). The proteins were electrophoretically transferred from gel to PVDF membranes. Prior to the transference, the membranes were blocked for 2 h at room temperature (RT) with 5% non-fat dry milk in PBST (phosphate-buffered saline, 0.05% Tween-20) followed by the incubation with the first antibody, goat IgG anti-beta conglutin (dilution 1:2000), overnight at 4 °C in continuous agitation. After washing 5 times with PBST, the membrane was incubated with secondary antibody goat anti-IgG rabbit conjugated with horseradish peroxidase (dilution 1:10,000) in 2% non-fat dry milk in PBST for 2 h at RT. The membrane was washed 5 times with PBST and the chemiluminescence signal was developed by membrane incubation with ECL Plus chemiluminescence substrate following the manufacturer’s instructions (BioRad). The light signal was detected by exposure of the membrane to C-Digit Blot Scanner (LI-COR).

### 2.11. ELISA Test for the Detection and Quantification of β-Conglutin Allergen Proteins in Lupin-Containing Food

The protein standards used for Lup an 1 identification and quantification was performed using purified allergen β-conglutin proteins. Coating the wells was performed by using purified conglutin β1 (50 μg). The plate wells containing purified proteins were used as blanks (controls without β-conglutin protein) and were simultaneously incubated (triplicate samples) overnight at 4 °C. The wells on the plates were washed 5 times with 200 μL of PBS for each well. In order to avoid the unspecific protein-binding sites, the samples were blocked in the coated wells by adding 200 μL blocking buffer (5% non-fat dry milk/PBS) per well, and incubated for 2 h at RT. The wells of the plates were then washed five times with 200 μL PBS. The first anti-IgG β-conglutin antibody (dilution 1:1000) was incubated in each well for 2 h at RT. The solution containing the antibody was removed and each well was washed five times with 200 μL PBS. The solutions on each well containing washing buffer were eliminated by flicking the plate over. The remaining drops on each well were eliminated by patting the plate with a paper towel. The wells on the plates were then incubated with a goat anti-IgG rabbit HRP conjugated antibody. The incubations were developed for 1 h and 30 min at RT, and the washes were performed five times with 200 μL PBS on each well. The development of the signal was made using a compatible substrate, 100 μL of TMB which was added to each well and incubated for 5 min at RT. The reaction was stopped using 1M Sulphuric Acid for 10 min. The signal was read in a microplate readed iMak (Bio-Rad) at 450 nm.

Protein detection and quantification following this ELISA method was performed in identical conditions as described above using protein extracts obtained from natural and processed food samples, and the extraction buffer alone for control samples.

## 3. Results and Discussion

### 3.1. Conglutin β1 Protein Structure Modelling and Antigenicity Assessment

**Figure 1:**
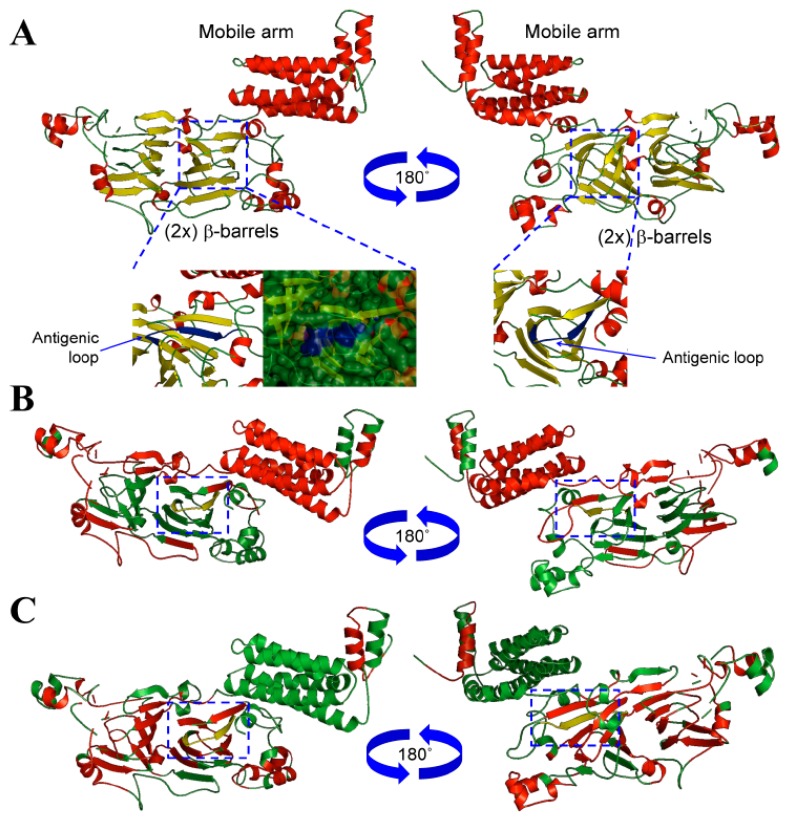
Structural and antigenic analysis of conglutin β1. (**A**) Three-dimensional structure of NLL conglutin β1 (Uniprot accession number F5B8V9) showing the mobile arm and globular domains. The structures were depicted as a cartoon diagram integrated by α-helices, β-sheets and coils (red, yellow and green colour, respectively). Two views (rotated 180 around the *x*-axis) are provided together with detailed views (carton diagram and surface) of the domain integrated by a coil-α-helix structure (blue colour), where the antigenic peptide chosen to develop the anti-β-conglutin antibody is located. (**B**) Three-dimensional structure depicted as a cartoon diagram of NLL conglutin β1 showing the antigenic β-cell sequential epitope regions (red colour), where the domain integrated by a coil-α-helix structure (yellow colour) are included. Two views (rotated 180 around the *x*-axis) are provided. (**C**) Three-dimensional structure depicted as a cartoon diagram of NLL conglutin β1 showing the Kolaskar and Tongaonkar antigenicity regions (red colour), where the domain integrated by a coil-α-helix structure (yellow colour) are also included. Two views (rotated 180 around the *x*-axis) are provided.

The NLL β-conglutin isoform sequences were retrieved from Uniprot database and an alignment was made to analyze the sequence features (Supplementary Figure S1). The length of these sequences ranged from 580 to 637 amino acids, and the variability was calculated (Figure S1), finding sequences with 77.4 to 97.8% of identity. The N-terminal sequence corresponding with the first 200 amino acids comprises a mobile arm (Figure S2), showing the highest variability among the seven isoforms. A globular structure was built for the rest of the β-conglutin sequence integrated by 2 β-barrels of antiparallel β-sheets (Figure 1) with large conserved areas, where it was included in the sequence used to make the synthetic peptide (Figure S1) and the anti-β-conglutin antibody used in this ELISA method. 

The crystallographic structures of particular seed storage proteins were used as templates to facilitate the correlative study between structure-functionality and antigenicity of these proteins [34]. To the best of the author’s knowledge, epitopes characterization through the 2-D and 3D comparative analysis of different members of vicilin protein family have been only performed in a few organisms, such as peanut [35], or lupin [21,36]. Furthermore, the use of computational homology modelling has allowed this study to uncover particular 3D structure features for the antigenic epitope belonging to this family of proteins (Figure 1A). In order to uncover these epitopic regions, an accurate modelling of conglutin β1 protein sequence was achieved following a work-flow, where best structural templates were used to build each domain of this protein. After obtaining a first model, a refinement process, including energy minimization and structural discordances were corrected [37].

A structural assessment for conglutin β1 model accuracy was performed to improve the stereo-chemical and energy minimization following a comparative analysis with the proteins’ templates (PDBs accession numbers 1uij, 2phl, 3s7e, and 2eaa, respectively).

The analysis of the templates showed z-values (normalized QMEAN4 scores) of −0.17, −0.56, −2.12 and 0.11, respectively, for the Q-mean parameter and −4.08 for the conglutin β1 built model, displaying comparative good z-scores. The overall quality of the structures was assessed using ProSA, showing a z-score of −5.31 for conglutin β1 model and −6.17, −6.2, −6.50, and −5.68 respectively for the individual crystallographic structural templates. Both, Q-mean and ProSA parameters showed comparable values between the conglutin β1 model and the PDB structure templates, which validated the accuracy and structure quality of the conglutin β1 protein model [38].

Therefore, a stereochemistry analysis was developed using Procheck based on the Ramachandran plot showing that the template models contained 92.2%, 92.9%, 94.8%, and 95.6%, respectively, of their residues located in favourable regions; 6.8, 6.6, 5.0, and 4.1, respectively, in allowed regions; 0.9, 0.6, 0.1 and 0.4, respectively, in generally allowed regions; and 0, 0, 0 and 0%, respectively, in disallowed regions. These values calculated for the conglutin β1 model showed 91.3%, 7.0%, 1.7%, 0%, respectively, finding even more residues located in favourable regions, less residues in allowed regions, and a similar number of residues in generally allowed and non-favourable regions. These parameters confirm the accuracy and reliability of the structural models built for the conglutin β1 protein sequence [39].

The antigenicity study of the β-conglutin proteins was made in order to identify one of the best common shared sequences on the protein with high antigenicity to be used for a synthetic peptide synthesis and further, for anti-β-conglutin antibody development. Bioinformatics tools were used based on biochemical principles as hydrophilicity/hydrophobicity, polarity and the volume of side changes, the antigenicity index, and B-cell epitopes identification. A semi-empirical method based on the physicochemical properties of amino acids and their frequencies of occurrence in experimentally known segmental epitopes was also used to identify the best antigenic sequence to develop the anti-β-conglutin antibody. Figure S1 highlights the antigenic peptide Nt-VDEGEGNYELVGIR-Ct chosen for experimental animal immunization. Figure S2 shows how this peptide has a combination of hydrophilic and hydrophobic amino acids (Kyte-Doolitle, Hope and Woods scales), which is one of the requirements to be an immunogenic peptide [40], constituting a high antigenicity sequence, where most of the amino acids are accessible to the solvent (Figure S2). In addition, this sequence integrates a structural loop (Figure S2), 2-D structure exerting good antigenic properties in a protein [41]. Furthermore, Figure 1B, Figures S1 and S2 show how the peptide chosen in this study corresponds to a sequential β-cell epitope with a high antigenicity index (Figure 1B,C) [40].

### 3.2. Purification and Reactivity of Conglutin β1 Protein

**Figure 2:**
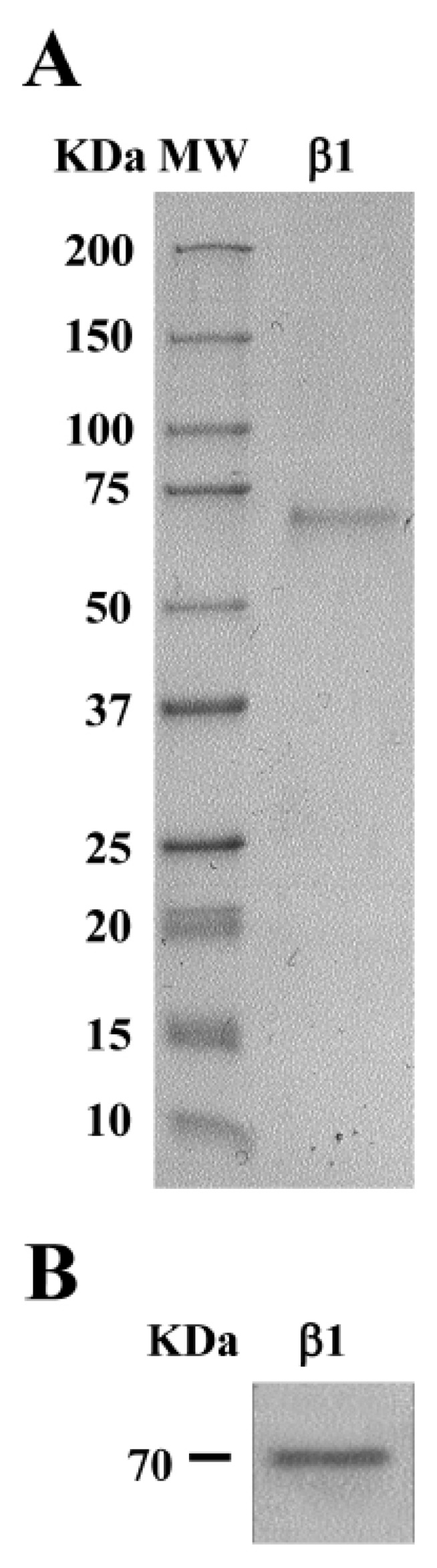
Purification of lupin main allergen β-conglutin (Lup an 1) protein. (**A**) The Coomassie-stained shows the purified β1-conglutin protein. (**B**) Immunoblotting shows the purified β1-conglutin protein identified by the anti-β-conglutin antibody. MW, molecular weight standard (kDa).

The purification of Lup an 1 was accomplished following the protocol set up by Jimenez-Lopez et al. [42]. Protein elution was accomplished with a linear gradient of imidazole (25–350 mM). The SDS–PAGE analysis of the eluted fractions showed a single protein band of ~70 kDa (Figure 2A), being the level of purity higher than 95%, with a typical yield of 27 mg/mL. An analysis by immunoblotting using the anti-β-conglutin protein antibody confirmed the identity of the purified β-conglutin protein in a unique reactive band (Figure 2B).

**Figure 3:**
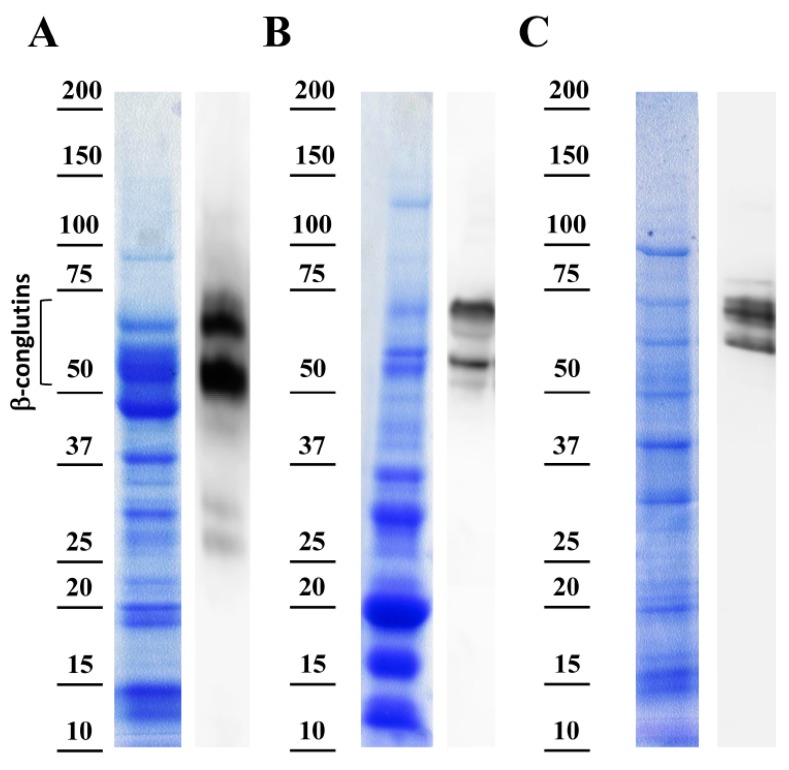
Specificity assessment of anti-β-conglutin antibody. Left panels show the Coomassie-stained SDS-PAGE protein bands from the total protein extracts, and the right panels show immunoblotting with the presence of β-conglutin proteins from (**A**) *Lupinus angustifolius*, (**B**) *Lupins albus*, and (**C**) *Lupinus lotus*.

The protein extracts were analyzed, as well as the specificity of the anti-β-conglutin antibody reactivity by using the most commonly used species of the sweet lupin group (*L. albus*, *L. luteus* and *L. angustifolius*) to make foodstuffs. This study examined the differences in the protein profiles and composition coming from these lupin species, where the protein extracts were extracted from mature seeds, and showing proteins separated in SDS-PAGE and visualized with Coomassie Brilliant Blue staining (Figure 3, left panels).

Overall, in the three species lupin extracts, the proteins with molecular weights from ~12 to 75 kDa were present in different ratios depending on the lupin specie (Figure 3A–C). No significant amount of proteins was detected above 75 kDa, just a prominent band of approximately 100 kDa. Based on the abundance of the seed proteins, four main groups of polypeptides were able to be identified: group 1— 75 kDa and above; group 2—from 45 to 75 kDa; group 3—from 25 to 45 kDa; and group 4—from 10 to 25 kDa.

Interestingly, *L. angustifolius* exhibited noticeable changes in the polypeptide composition between 50 and 75 kDa (Figure 2A). However, the three species displayed differential polypeptides content in this range of MW. These proteins localized in the expected size range (~70 kDa) related to β-conglutin polypeptides are present in the seeds from mature seeds onward [43] in *L. angustifolius*, *L. albus* and *L. luteus*, and particularly identified as these proteins obtained in the current study by the over-expression and purification (Lup an 1, F5B8V9, with theoretical MW ~70 kDa) (Figure S1). The major differences in polypeptides composition and abundance were observed in the range of MW concerning 25–45 kDa. On the other hand, protein profiles present in the range of 10–25 kDa were similar among the entire lupin species analyzed. 

The right panels in Figure 3A–C showed polypeptide bands corresponding to β-conglutin by immunoblot assays using the anti-β-conglutin antibody, which is able to recognize the seven β-conglutin isoforms from *L. angustifolius* and the recently identified sequence of *L. albus* β-conglutin (Uniprot accession number Q6EBC1). This antibody denoted a high specificity since it showed specific binding to Lup an 1 while no signal from reactive bands was obtained in the control assays using pre-immune serum. In the present study, and due to the comparable proteolytic processing of β-conglutin proteins in mature seeds from the three different lupin species [44], the presence of comparable reactive bands were found in these three species using the anti-β-conglutin antibody, in the range of 50 to 75 kDa (Figure 3). This corresponds to the mature forms of β-conglutin and shows the high specificity of this antibody to be used in the ELISA method, and other potential related applications. Indeed, the ELISA method developed in this study positively detected and identified the lupin main β-conglutin allergen on each of the different lupin species. It shows the suitability of this anti-β-conglutin antibody to be used in the ELISA method for the identification of Lup an 1 in foodstuffs, independently of the proteins composition in these three species contained in foodstuffs. The identification and quantification of Lup an 1 using the antibody varied depending on the specie analyzed, where most abundantly detected (574.7918 ± 19.887 ng) in *L. angustifolius* seed protein extract, followed by *L. luteus* (457.8474 ± 15.8272 ng), and *L. albus* (299.3749 ± 19.7757 ng) (Table 2). These results are comparatively in agreement with β-conglutin protein levels found in these species [7].

### 3.3. β-Conglutin Proteins Main Allergen Detection Capability in Lupin—Derived Products

The analytical methods capable to detect, identify and quantify the main lupin allergen protein in food with high specificity and sensitivity are of great importance to help the food industry in allergen management, and to guarantee the life quality of sensitized/allergic individuals. Although ELISA is a frequently used technique for the detection and identification of food allergen proteins, there is no currently available ELISA test to detect the seed allergen proteins in all three most common lupin species (*L. angustifolius*, *L. albus*, and *L. luteus*) used for foodstuff production. For this reason, an ELISA newly developed and assessed method capable of detecting and detecting the presence of the lupin main allergen β-conglutin protein (Lup an 1) in natural and foodstuffs samples containing these three lupin species which constitutes a molecular tool of great interest. In the present study, a broad range of food has been analyzed. Labelling information of lupin flour analyzed in the current study had non-defined specie composition. In addition to the three lupin species most commonly used in food and feed industry, this study analyzed non lupin based products such as peanut butter; legumes as chickpea, lentils, or faba beans; wheat toasted bread (negative control); other product that may content lupin traces as gluten free chocolate biscuits; and cooked food (roasted, boiling, heating in oven products) which may be an indicative of how this method can be implemented in processed (cooked) food; as well as fermented or pickle foodstuff; lupin milk and cheese; meat; along with positive and negative control foodstuffs. The data from the quantification of Lup an 1 has been summarized in Table 1. These samples were analyzed in order to check the suitability of the designed antibody (anti-β-conglutin) to quantify the main allergen β-conglutin from protein extracts obtained following a specific protocol for vicilin (β-conglutin) proteins extraction, being these proteins specifically soluble in solutions containing NaCl [33]. 

Some methods have been previously reported for the detection of lupin proteins in foodstuffs by enzyme-linked immunosorbent assay (ELISA) [4,45,46,47]. However, they exhibited main disadvantages in comparison to the current study:

(1) The protein extraction protocol in the current study is highly specific for the extraction of the vicilin family of proteins (β-conglutins), based on the presence of NaCl (0.25M) in the extraction buffer [33]. Previous studies made protein extractions using general protein extraction buffers [4,45,47], or using alternative methods from commercial kits [48] currently no longer available (Abnova, http://www.abnova.com/products/products_detail.asp?catalog_id=KA3310), displaying very limited or no information about: (i) The antibody design; (ii) antibody production and use in the ELISA detection method; (iii) the limited information about protocol for total proteins extraction, which may not be specific for β-conglutin extraction; (iv) no information about lupin species used [46,48] to obtain this protein extract. The last two are the main factors with high impact in the protein extract characteristics, such as a low amount or not of Lup an 1 content. These disadvantages may result in the increase of the number of false positives as a result of the detection of non-allergen proteins from a low specific antibody, or using non-appropriate protein extracts.
(2) A second advantage of the current method compared with previous ones and commercial kits is the design and the production of the antibody (anti-IgG β-conglutin proteins). In the current study, the experimental animal was immunized with a synthetic peptide commonly shared by the seven NLL β-conglutin protein isoforms. This synthetic peptide constitutes a highly antigenic epitope in these proteins probed in the current study, while also exhibits a high specificity to detect the lupin main allergen Lup an 1 in the most frequently used lupin species. On the contrary, previous methods [4,45,46,47] have used the whole crude protein extract from lupin flour to immunize the experimental animal and obtain the antibody. The method to produce this antibody makes this antibody non-specific, and detecting a wide range of proteins, including many non-allergenic proteins, may lead to false positive detections. The detection of false positives might be also enhanced due to the implementation of a buffer inappropriate for vicilin protein extraction from lupin-derived foodstuffs.
(3) The current method exhibited another advantage which was the type of standards (Figure S3) that was made for a specific quantification of Lup an 1. Previous methodological developments of standards for the quantification were based on lupin flour total protein extract [4,45,46,47]. This may induce variable immunization for a complex mix of proteins (allergenic and non-allergenic proteins) from the crude extract leading to an excess or lack of reactivity in the standard samples because a variable representation or content of Lup an 1 (over or under representation, depending on the extraction method).

Therefore, lupin allergy reactions could be developed with high severity from primary sensitization to lupin proteins or due to the cross-reactivity with proteins from other legumes. The abundance of these proteins in many (natural or processed) foodstuffs has led to the European Union (EU Regulation No. 1169/2011) [49] to include lupin in a list of allergens with mandatory identification in all lupin containing foodstuffs. Indeed, an EU Labelling Directive involves the mandatory declaration of manufacturers regarding the presence of 14 allergenic products on pre-packaged foods [50]. Since lupin is included among these, analytical tools should be developed for the detection of lupin allergen traces in complex and processed foodstuffs. Thus, a high accurate method to detect and identify the lupin main allergen in food would be very valuable, with a particular importance due to the increasing prevalence of lupin allergy [16] among the population. The current study used highly pure (>95%) recombinant purified Lup an 1 (Figure 2), with the combination of an antibody that was developed using a specific synthetic peptide (main antigenic epitopes of the Lup an 1), while making the identification and quantification of the Lup an 1 highly specific and accurate. 

In order to evaluate the application of the developed method to actual foodstuffs, several commercial samples labelled as “it may contain traces”, “with or without lupine” were tested for the presence of Lup an 1. The summarized ELISA results, together with the corresponding label information of samples, are described in Table 1 and Table 2. 

The Lup an 1 lower content value in the food samples analyzed was 0.0406 ± 0.0009 ppm for toasted bread (Table 2). This result is in the comparable range of an ELISA method developed to detect soy protein content in foodstuff [51]. In this regard, the lowest eliciting dose for allergic reactions to lupin, responsible for inducing mild symptoms in peanut-sensitized patients, was 0.5 mg of lupin flour [17]. More recently, the VITAL program of the Allergen Bureau of Australia and New Zealand (ABA) established 4 mg of protein as the reference allergenic dose for lupin [52]. Taking into consideration that conglutins are the most abundant protein in lupin, being 40% of the protein seeds content [7], our ELISA method is by far capable of detecting these allergenic reference doses of lupin proteins in foodstuffs as demonstrated in this study. 

Overall, NLL exhibited the highest Lup an 1 value (574.7918 ± 19.8876 ng) compared to all the samples investigated. The analysis of the sample number 2 (biscuits), and these samples declaring the information “may contain traces of lupine”, showed no Lup an 1 content (Table 2), which might be due to non-contamination with lupin flour or other lupin derived component. Despite this fact, the manufacturer has implemented the common practice of the precautionary labelling. The same result was obtained from samples as number 11 (Table 1 and Table 2) labelled as “non lupin content”.

The estimated content of Lup an 1 in commercial lupin flour displayed an intermediate value among the three lupin seed species analyzed (Table 1 and Table 2), which is in agreement with the labelling composition as sweet lupin attributed to three main domesticated lupin species (*L. albus*, *L. luteus* and *L. angustifolius*). The samples number 5, 9, 10, and 12 to 16 containing “lupine flour” or “lupine protein” among their ingredients, Lup an 1 was detected, identified and quantified in a range of allergen in accordance with their labelling (Table 1 and Table 2) and also, to the variable quantity of seed flour from these three different sweet lupin species.

### 3.4. Specificity of the Anti-β-Conglutin Antibody Tested in Processed Foodstuffs and Potential Cross-Reactive

Lupin allergen proteins have been identified to be stable towards thermal treatment [5,47] in studies concerning the impact of the type of food processing on the allergenicity. Indeed, comparable results have been obtained in the current study for soy and chickpea-containing products, as well as for food processed under fermentation, soaking, extrusion, cooking, boiling and microwave heating conditions. These cooking processes do not affect the allergenic potential of protein extracts, while the autoclaving process drastically reduces the binding capacity of antibodies tested with serum from atopic patients [53,54,55]. This may be due to the conformational epitopes disappearing in these proteins under these processed conditions. 

The main factors with a significant influence in the allergen analysis with ELISA methods are food matrices, the level of food processing and the ELISA test kit selection [56]. The current study analyzed the different foodstuffs (Table 1 and Table 2), which were differentially processed. The ELISA method was able to detect, specifically identify and quantify Lup an 1 allergen: toasted [bread (8.1250 ± 0.1701 ng)], fermented [Lupinen—Tempeh (152.9862 ± 7.3353 ng)], Pickle [Pickled lupin (293.5833 ± 11.0853 ng)], Pasteurized [Lupinen drink (59.8660 ± 0.8389 ng)], and cooked [BOLOGNESE SAUCE (3.8890 ± 0.2599 ng)] foodstuff. Furthermore, a previous study indicated that boiling affected the allergenicity of legumes, particularly soy (Alvarez-Alvarez et al., 2005). Despite this fact, this study was able to detect and quantify Lup an 1 in boiled lupin based foodstuffs like Lupinen-tempeh (152.9862 ± 7.3353 ng) and LupinenBurger-Mediterranean (129.1667 ± 3.6839 ng). 

Allergy reactions to lupin seed proteins are often triggered in atopic patients sensitized to proteins from other legumes, such as pea, lentil, soya, chickpea and peanut [5,21,57]. These allergy reactions arise against seed storage proteins from the Leguminosae family included in foodstuffs, since these proteins share similar epitopic regions in these different legume seed proteins. In this regard, atopic patients sensitized to one legume allergen protein might develop cross-reactions to proteins from another legume [58,59]. The most frequently described and important cross-reactivity from a clinical point of view is between lupin and peanuts [5]. Additional cross-reactivity between lupin and other legumes (lentil, pea) has been also reported, but in low percentages in children [5,57]. Furthermore, at a molecular level, lupin IgE cross-reactivity has been reported for peanut, soya, lentil, chickpea and bean [5]: Lupin sensitized patients were reported to develop cross-reactivity between 59–72% [13,20,60] and 52–55% [20,60] for soya bean and pea, respectively. Allergy reactions to peanuts are currently the most frequently identified legume allergy, followed by soya bean [57]. 

In this regard, a high specific and reliable method to detect the presence of other legume proteins is of crucial importance in order to avoid the development of cross-reactivity allergy reactions. The method developed in the current study was able to detect the lupin main allergen Lup an 1 with high specificity compared to other previous methods. However, the method may show false negative results as a consequence of the low antibody specificity and/or the absence of detection of allergen proteins that were not extracted in enough quantity by these alternative previously developed methods. On the other hand, false positive results as consequence of the use of a non-high specific antibody developed against a whole protein extract may not have clinical relevance. This may further lead to cross-reactivity between the antibody and the target protein from a related species with similar detection results (lack of specificity), and in the end, this scenario has notable consequences for the quality of life of people.

In the current study, lupin cross-reactivity with other legumes could be clinically relevant. For that reason, the authors developed an accurate, reliable, and highly specific method to detect, identify and quantify the main lupin allergen Lup an 1 that may be responsible of cross-reactivity with other legume proteins [11,21,22,36]. The high specificity of our anti-β-conglutin antibody is capable of avoiding false positives as a result of the cross-reactivity (Table 2). An analysis of different food products containing multiple legume proteins, such as peanut butter, chickpea, lentil, faba bean, even cereals resulted in the negative detection of homologous proteins to Lup an 1. In comparison, other ELISA methods previously developed showed low specificity of their antibodies used in the ELISA methods, since cross-reactivity showed with other legumes: Either with pea, chickpea, peanut, lentil, and soy [45], with brown bean and fenugreek (Holden et al., 2007); black bean and soy [46]; or even with non-legume proteins such as sunflower seed, cashew, almond, and pumpkin seed [61]. Furthermore, Koeberl et al. [48] used three available commercial kits to analyze cross-reactivity between lupin and other legume proteins finding that all peanut samples tested showed cross-reactivity on ELISA test kit B and C [48]. In addition, cross-reactivity was also described for the entire lentil samples analyzed, thus promoting false positive results in all analyzed legume samples. These results highlight the importance of transparency in the information provided by developed kits, at least in the characteristics of ELISA antibodies and protocols for their production, and further for the proteins extraction protocols implemented in these kits. This could lead to more uncertainty about the link between the food allergen protein identification and quantification, with the development of clinical therapies. 

## 4. Conclusions

A newly developed ELISA assay was assessed for its capability to detect, identify and quantify the lupin main allergen β-conglutin protein (Lup an 1) in natural products and processed foodstuffs (toasted, boiled, fermented, cooked, pickled, pasteurized lupin-based products). Cross-reactivity was tested using peanut and other legumes, obtaining negative results. 

The standards were made using recombinant purified β-conglutin proteins, showing a highly specific method for particular allergen proteins compared to previous developments. This fact highlights the importance of particular molecular tools to develop a more reliable and highly specific analytical method for food allergen protein detection. This recombinant protein could constitute an available improvement for the quantification of food allergens, which should be approved as (certified) reference material for food allergens identification helping to implement the labelling mandatory EU regulations and as relevant proteins for clinical allergy research. 

Finally, this study demonstrated that this ELISA method is based on accurate and reliable molecular tools, which can contribute to a more effective management of allergens by the food industry, the regulatory agencies and clinicians, thus helping to protect the health of sensitized/allergic consumers.

## Figures and Tables

**Table 1:** Summary of samples analyzed in this study. The information includes brand, the lupin content, and the food processing state.

Product Number	Product	Product Information Related to Lupin Content	Web
1	Toasted bread-Crostini	Lupin protein	https://www.schaer.com/en-int/p/crostini
2	Gluten Free Maxi Sorrisi Chocolate Biscuit	It may content traces of lupin	https://www.schaer.com/en-int/p/maxi-sorrisi
3	Commercial lupin flour	Sweet lupin flour	https://www.arche-naturkueche.de/de/produkte/europaeische-kueche/backen-binden/s%C3%BCsslupinenmehl
4	Peanut butter	Roasted peanuts (97%), palm oil, and sea salt.	https://shop.wholeearthfoods.com/collections/award-winning-nut-butters/products/whole-earth-dark-roasted-peanut-butter-340-g
5	Carob spread hazelnut Chocolate duo	Lupin flour 5%	https://greenest.ee/en/product/carobella-carobella-chocolate-duo-bio-350-g
6	Seeds (*Lupinus albus* L.)	Seeds	https://www.semillascantueso.com/
7	Seeds (*Lupinus luteus* L.)	Seeds	https://www.semillascantueso.com/
8	Seeds (*Lupinus angustifolius* L.)	Seeds	https://www.semillascantueso.com/
9	Lupinen BOLOGNESE SAUCE	Sweet lupin seeds cooked (8%)	https://www.veggie-shop24.com/food/ready-meals/sauces-and-dips/alberts-lupine-bolognese-sauce-organic-300g
10	Fresh spread cheese	Lupin protein (6.6%)	https://www.veggie-shop24.com/food/cheese-alternatives/cream-cheese/made-with-luve-frisch-cremiges-streichglueck-fresh-creamy-spread-bliss-herbs-150g
11	Wheat toasted bread	Non lupin content	http://www.bimbo.es/productos/tostados#asies
12	Lupinen—Tempeh LUPEH	Boiled sweet lupin seeds 99%	https://www.veggie-shop24.com/food/vegan-basics/tofu-and-tempeh/alberts-lupeh-lupine-tempeh-organic-170g
13	Lupinen BURGER—MEDITERRANEAN	Boiled sweet lupin seeds 15%	https://www.veggie-shop24.com/food/meat-alternatives/burger-and-grill/alberts-lupine-burger-gluten-free-organic-200g
14	Pickled lupine	Lupin (*L. albus*)	https://es.openfoodfacts.org/producto/8480000330987/altramuces-encurtidos-hacendado
15	Lupinen Drink	Lupin protein (2.3%)	https://www.alles-vegetarisch.de/lebensmittel/milchalternativen-und-desserts/milchersatz/made-with-luve-lupinen-drink-natur-1l
16	TOFU smoked	Made with soya bean. Non lupin content.	https://www.veggie-shop24.com/food/vegan-basics/tofu-and-tempeh/alberts-tofu-smoked-organic-1kg
17	Boiled lentils	Non lupin content	
18	Boiled chickpea	Non lupin content	https://www.deliberry.com/mercadonamadrid/alimentacion-general/legumbres-y-verduras-envasadas
19	Boiled faba bean	Non lupin content	https://www.deliberry.com/mercadonamadrid/alimentacion-general/legumbres-y-verduras-envasadas

**Table 2:** Detection and quantification of the NLL main seed allergen β-conglutin (Lup an 1) in natural and processed food samples. Each number corresponds to the following food stuff: 1: toasted bread; 2: biscuit; 3: lupin flour; 4: peanut butter; 5: carob spread; 6: white lupin seed; 7: yellow lupin seed; 8: blue lupin seed; 9: sauce; 10: fresh cheese; 11: wheat bread, 12: fermented food; 13: meat; 14: pickled lupine; 15: lupin drink; 16: Soy (Smoked TOFU); 17: Lentils; 18: chickpea; 19: Faba bean.

Natural/Food Samples	Absorbance (450 nm) ^1^	Allergen β-Conglutin Proteins Quantity (ng) (Diluted Samples)	Allergen β-Conglutin Proteins Final Quantity (ng)	Allergen β-Conglutin Proteins Quantification (ppm)
1	0.0390	2.0313	8.1250 ± 0.1701	0.0406 ± 0.0009
	0.0380	1.9792		
	0.0400	2.0833		
2	0	0	0	0
	0	0		
	0	0		
3	1.8430	95.9896	391.8753 ± 11.1958	1.9594 ± 0.0560
	1.8430	95.9896		
	1.9570	101.9271		
4	0	0	0	0
	0	0		
	0	0		
5	0.3890	20.2604	80.9028 ± 0.3541	0.4045 ± 0.0018
	0.3860	20.1042		
	0.3900	20.3125		
6	1.3430	69.9479	299.3749 ± 19.7757	1.4969 ± 0.0989
	1.5670	81.6145		
	1.4010	72.9688		
7	2.2890	119.2188	457.8474 ± 15.8272	2.2892 ± 0.0791
	2.1030	109.5313		
	2.2010	114.6354		
8	2.8940	150.7292	574.7918 ± 19.8876	2.8740 ± 0.0994
	2.6910	140.1563		
	2.6920	140.2083		
9	0.0190	0.9896	3.8890 ± 0.2599	0.0194 ± 0.0013
	0.0200	1.0417		
	0.0170	0.8854		
10	1.0340	53.8542	216.1112 ± 0.5197	1.0806 ± 0.0026
	1.0380	54.0625		
	1.0400	54.1667		
11	0	0	0	0
	0	0		
	0	0		
12	0.7840	40.8334	152.9862 ± 7.3353	0.7649 ± 0.0367
	0.7060	36.7708		
	0.7130	37.1354		
13	0.6430	33.4896	129.1667 ± 3.6839	0.6458 ± 0.0184
	0.6000	31.2500		
	0.6170	32.1354		
14	1.4020	73.2105	293.5833 ±11.0853	1.4252 ± 0.0554
	1.5140	77.5442		
	1.3860	70.8534		
15	0.2840	14.7917	59.8660 ± 0.8389	0.2993 ± 0.0042
	0.2930	15.2604		
	0.2850	14.8438		
16	0	0	0	0
	0	0		
	0	0		
17	0	0	0	0
	0	0		
	0	0		
18	0	0	0	0
	0	0		
	0	0		
19	0	0	0	0
	0	0		
	0	0		

^1^ Triplicated experiments.

## Supplementary Materials

The following are available online at https://www.mdpi.com/2304-8158/8/10/513/s1, Figure S1: Multiple alignment of the deduced amino acid sequences of β1 to β7., Figure S2: Antigenicity assessment of conglutin β1., Figure S3: Standard curve made of the conglutin β1 protein.
