# Supplementary material for: Narrow-Leafed Lupin Main Allergen β-Conglutin (Lup an 1) Detection and Quantification Assessment in Natural and Processed Foods
Source: Foods. 2019 Oct 18;8(10):513. doi: 10.3390/foods8100513 (PMC6835513; doi:10.3390/foods8100513)
Supplement: Supplementary file 1 [file foods-08-00513-s001.zip › Figure S1.pdf]

```
                   5         15        25        35        45        55        65        75        85        95        105       115
               ....|....|....|....|....|....|....|....|....|....|....|....|....|....|....|....|....|....|....|....|....|....|....|....|
F5B8W2_beta4   MIKMRVRFPTLVLLLGIVFLMAVSIGIAYGEKNVIKNHERPQEREQEERDPRQQPRPHHQEEQEREHRREEERDREPSRGRSESEESREEEREQRREPRREREQEQQPQHGRREEEEE--
F5B8W4_beta6   MIKMRVRFPTLVLLLGIVFLMAVSIGIAYGEKNVIKNHERPQEREQEERDPRQQPRPHHQEEQEREHRREEERDREPSRGRRESEESREEEREQRREPRREREQEQQPQHGRREEEEE--
F5B8W1_beta3   MAKMRVRFPTLVLLLGIVFLMAVSIGIAYGEKNVLKNHERPQEREQEERDPRQQPRPHHQEEQEREHRRE------------SEESQEEEREQRREPRREREQEQQPQHGRREEEEE--
F5B8W0_beta2   MANMRVKFPTLVLLLGIVFLMAVSIGIAYGEKNAIKNHERPQEREQEERDPRQQPRPRHQEEQEREHGREEERNREPSRGRSESEESREEEREQRREPSRGREQEQQPQHGRREEEEE--
F5B8W3_beta5   MAKMRVRFPMLVLLLGVVFLLAVSIGIAYGEKDVIKNPERPEERQEEERDPRQPPRSRQQEEQEREHRREKERDREPSRGRSESKQSQEEERERRKEHDREREQEQQPQYGRRHEEEEKG
F5B8W5_beta7   MARMRVRFPTLVLLLGILFLMAVSIGIAYGEKDVIKNHERPGEREHEERDPRQQPRPRKQEEQEREHRREEEHDRDPSRGRRESEERQEEERERRREPCREREQEQQPQHGRREEEEE--
F5B8V9_beta1   MAKMRVRLPMLILLLGVVFLLAASIGIAYGEKDFTKNP--PKEREEEEHEPRQQPRPRQQEEQEREHRREEKHDGEPSRGRSQSEESQEEEHERRREHHREREQEQQPRPQRRQEEEEEE
Consensus      * .***::* *:****::**:*.*********:  **   * **:.**::*** **.::******** **            *:: :***:*:*:*  * *******:  **.****

                   125       135       145       155       165       175       185       195       205       215       225       235
               ....|....|....|....|....|....|....|....|....|....|....|....|....|....|....|....|....|....|....|....|....|....|....|....|
F5B8W2_beta4   -----WQPRRQRPQSRREEREQEQGSSSSSRRQSGYERRE---EREQE--------------------------------QEQGSRSDSRRQRNPYYFSSERFQTLYRNRNGQIRVLER
F5B8W4_beta6   -----WQPRRQRPQSRREEREQEQGSSSSSRRQSAYERREQREEREQE--------------------------------QEQGSRSDSRRQRNPYYFSSERFQTLYRNRNGQIRVLER
F5B8W1_beta3   -----WQPRRQRPQSRREEREQEQGSSSSSRRQSGYERREQREEREQE--------------------------------QEQGSRSDSRRQRNPYYFSSERFQTLYRNRNGQIRVLER
F5B8W0_beta2   -----WQPRRQRPQSRREEREQEQGSSSSSGRQSGYERREQREEREQQ--------------------------------QEQDSRSESRRQRNPYYFSYERFQTLYKNRNGQIRVLER
F5B8W3_beta5   EEEEEGQARRQRPQRRREEREQEQGSSSESRRQSGDERRHRHEKREQREEREQEQGSSSGRQSDYGRRQRHEGREQREEREQEQGSSSESHRLRNPYYFSSERFQTRYKNKNGQIRVLER
F5B8W5_beta7   --EEEWQPRRLRPQSRKEEREQEQGSSSSSRKQSGYERRQYHERREQRDEKE----------------------------KEQDSRSDSRRQRNPYHFSSERFQTRYRNRNGQIRVLER
F5B8V9_beta1   EEWQPRRQRPQSRREEREEREQEQGSSSGSQRGSGDERRQHRERRVHREERE----------------------------QEQDSRSDSRRQRNPYHFSSNRFQTYYRNRNGQIRVLER
Consensus          : *    :  .:*********** * : *. ***.    .* :.                          :**.* *:*:* ****:** :**** *:*:*********

                   245       255       265       275       285       295       305       315       325       335       345       355
               ....|....|....|....|....|....|....|....|....|....|....|....|....|....|....|....|....|....|....|....|....|....|....|....|
F5B8W2_beta4   FDQRTDRLENLQNYRIVEFQSKPNTLILPKHSDADYILVVLNGSATITIVNPDKRQSYNLENGDALRLPAGTTSYILNPDDNQNLRVVKLAIPINNPGNFYDFYPSSSKDQQSYFSGFSR
F5B8W4_beta6   FDKRTDRLENLQNYRIVEFQSKPNTLILPKHSDADYILVVLNGSATITIVNPDKRQSYNLENGDALRLPAGTTSYILNPDDNQNLRVVKLAIPINNPGNFYDFYPSSSKDQQSYFSGFSR
F5B8W1_beta3   FDQRTNRLENLQNYRIVEFQSKPNTLILPKHSDADYILVVLNGSATITIVNPDKRQSYNLENGDALRLPAGTTSYILNPDDNQNLRVVKLAIPINNPGNFYDFYPSSSKDQQSYFSGFSK
F5B8W0_beta2   FDQRTNRLENLQNYRIVEFQSKPNTLILPKHSDADYILVVLNGRATITIVNPDKRQAYNLEHGDALRLPAGTTSYILNPDDNQNLRVVKLAIPINNPGNFYDFYPSSTKDQQSYFNGFSR
F5B8W3_beta5   FDQRTNRLENLQNYRIVEFQSRPNTLILPKHSDADYILVVLNGRATITIVNPDKRQAYNLEYGDALRLPAGTTSYILNPDDNQDLRVVKLAIPINNPGKFYDFYPSRTKDQQSYFSGFSK
F5B8W5_beta7   FDQRTNRLENLQNYRIVEFQSNPNTLILPKHSDADYILVVLNGRATITIVNPDKRQAYNLEYGDALRVPAGTTSYILNPDDNQNLRVVKLAIPINNPSNFYDFYPSSTKDQQSYFSGFSK
F5B8V9_beta1   FNQRTNRLENLQNYRIIEFQSKPNTLILPKHSDADFILVVLNGRATITIVNPDKRQVYNLEQGDALRLPAGTTSYILNPDDNQNLRVAKLAIPINNPGKLYDFYPSTTKDQQSYFSGFSK
Consensus      *::**:**********:****.*************:******* ************ **** *****:**************:***.*********.::****** :*******.***:

                   365       375       385       395       405       415       425       435       445       455       465       475
               ....|....|....|....|....|....|....|....|....|....|....|....|....|....|....|....|....|....|....|....|....|....|....|....|
F5B8W2_beta4   NTLEATFNTRYEEIQRILLGNEDEQEDDEQRHGQEQSHQDEGVIVRVSKEQVQELRKYAQSSSRKGKPSKSGPFNLRSNKPIYSNKFGNFYEITPNRNPQAQDLDISLTFIEINEGALLL
F5B8W4_beta6   NTLEATFNTRYEEIQRILLGNEDEQEDDEQRHGQEQSHQDEGVIVRVSKEQVQELRKYAQSSSRKGKPSKSGPFNLRSNKPIYSNKFGNFYEITPNRNPQAQDLDISLTFIEINEGALLL
F5B8W1_beta3   NTLEATFNTRYEEIQSILLGNEDEQEDDEQWHGQEQSHQDEGVIVRVSKEQVQELRKYAQSSSRKGKPYESGPFNLRSNKPIYSNKFGNFYEITPDRNPQAQDLDISLTFIEINEGALLL
F5B8W0_beta2   NTLEATFNTRYEEIQRIILGNEDGQEDEEQSRGQEQSHQDQGVIVRVSKEQIQELRKHAQSSSGKGKPSESGPFNLRSDEPIYSNKFGNFYEITPDRNPQAQDLDISLTFIEINEGGLLL
F5B8W3_beta5   NTLEATFNTHYEEIQRILLGYEDEQEDEEQRREQEQSHQDEGVIVRVSKEQIQELRKHAQSSSRKGKPSESGPFNLRSNEPIYSNKFGNFYEITPDRNPQVQDLDISLIFTEISEGALLL
F5B8W5_beta7   NTLEATFNTRYEEIQRILLGNEDEQEDEEQRRGQEQSYQDEGVIVRVSKEQIQELRKHAQSSSRKGKPSESGPFNLRSNESIYSNKFGNFYEITPERNPQVQDLDISLTFTEINEGALLL
F5B8V9_beta1   NTLEATFNTRYEEIERVLLGDDELQENEKQRRGQEQSHQDEGVIVRVSKKQIQELRKHAQSSSGEGKPSESGPFNLRSNKPIYSNKFGNFYEITPDINPQFQDLNISLTFTEINEGALLL
Consensus      *********:****: ::** :: **:::* : ****:**:********:*:*****:***** :*** :********::.**************: *** ***:*** * **.**.***

                   485       495       505       515       525       535       545       555       565       575       585       595
               ....|....|....|....|....|....|....|....|....|....|....|....|....|....|....|....|....|....|....|....|....|....|....|....|
F5B8W2_beta4   PHYNSKAIFVVLVDEGEGNYELVGIRDQQRQQDEQE---------VRRYSARLSEGDIFVIPAGHPISINASSNLRLLGFGINADENQRNFLAGSEDNVIRQLDTEVKGLTFPGSTEDVE
F5B8W4_beta6   PHYNSKAIFVVLVDEGEGNYELVGIRDQQRQQDEQE---------VRRYSARLSEGDIFVIPAGHPISINASSNFRLLGFGINADENQRNFLAGFEDNVIRQLDREVKGLTFPGFAEDVE
F5B8W1_beta3   PHYNSKAIFVVVVDEGEGNYELVGIRDQQRQQDEQE---------VRRYSARLSEGDIFVIPAGHPISINASSNLRLLGFGINADENQRNFLAGSEDNVIRQLDREVKGLIFPGSAEDVE
F5B8W0_beta2   PHYNSKAIFVVVVDEGEGNYELVGIRDQERQQDEQEQEE------VRRYNAKLSEGDIFVIPAGHPISINASSNLRLLGFGINADENQRNFLAGSEDNVIRQLDKEVKQLTFPGSVEDVE
F5B8W3_beta5   PHYNSKAIFVIVVDEGEGNYELVGIRNQQRQQDEQEVEE------VRSYNARLSEGDILVIPAGHPLSINASSNLRLLGFGINADENQRNFLAGSEDNVIRQLDREVKELIFPGSAEDVE
F5B8W5_beta7   PHYNSKAIFIVVVDEGEGNYELVGIRDQQRQQDEQEEEEE----EVRRYSARLSEGDIFVIPAGYPISVNASSNLRLLGFGINANENQRNFLAGSEDNVISQLDREVKELTFPGSAQDVE
F5B8V9_beta1   PHYNSKAIFIVVVDEGEGNYELVGIRDQQRQQDEQEEEYEQGEEEVRRYSDKLSKGDVFIIPAGHPLSINASSNLRLLGFGINANENQRNFLAGSEDNVIKQLDREVKELTFPGSIEDVE
Consensus      *********:::**************:*:*******         ** *. :**:**:::****:*:*:*****:*********:********* ***** *** *** * ***  :***
                        [red bar under positions ~493–506]

                   605       615       625       635       645
               ....|....|....|....|....|....|....|....|....|....|
F5B8W2_beta4   RLIKNQQQSYFANAQPQQQQQ-REREGRRGRRGHISSILSTLY-------
F5B8W4_beta6   RLIKNQQQSYFANAQPQQQQQ-REREGRHGRRGHIFSILSTLY-------
F5B8W1_beta3   RLIKNQQQSYFANAQPQQQQQ-REREGRHGRRGHISSILSTLY-------
F5B8W0_beta2   RLIKNQQQSYFANAQPQQQQQ-REKEGRRGRRGLSFPFRSLFTKLLSTIM
F5B8W3_beta5   RLIRNQQQSYFANAQPQQQQQQREKEGRRGRRGPISSILSALY-------
F5B8W5_beta7   RLIKNQQQSYFANAQPQQKQQ-REKEGRRGRRSLISSILSTLY-------
F5B8V9_beta1   RLIKNQQQSYFANAQPQQQQQ-REKEGRRGRRGPISSILNALY-------
Consensus      ***:**************:** **:***:***.    .: . :
```

Identity (%)	β1	β2	β3	β4	β5	β6	β7
β1	100.0	**77.4**	78.1	79.1	77.6	78.3	81.4
	β2	100.0	87.3	89.2	78.8	88.7	85.2
		β3	100.0	94.7	79.5	94.7	86.6
			β4	100.0	80.5	**97.8**	87.7
				β5	100.0	79.7	82.0
					β6	100.0	87.2
						β7	100.0
