# Supplementary figures and images for: Narrow-Leafed Lupin Main Allergen β-Conglutin (Lup an 1) Detection and Quantification Assessment in Natural and Processed Foods
Source: Foods. 2019 Oct 18;8(10):513. doi: 10.3390/foods8100513 (PMC6835513; doi:10.3390/foods8100513)

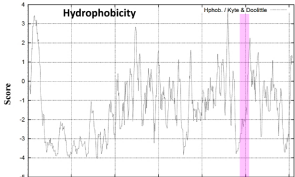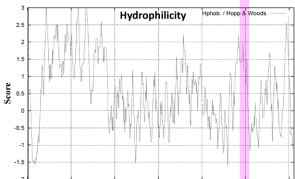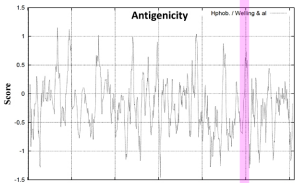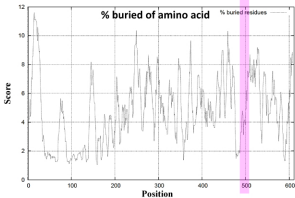

Supplement: Supplementary file 1 [file foods-08-00513-s001.zip › Figure S2.pdf]

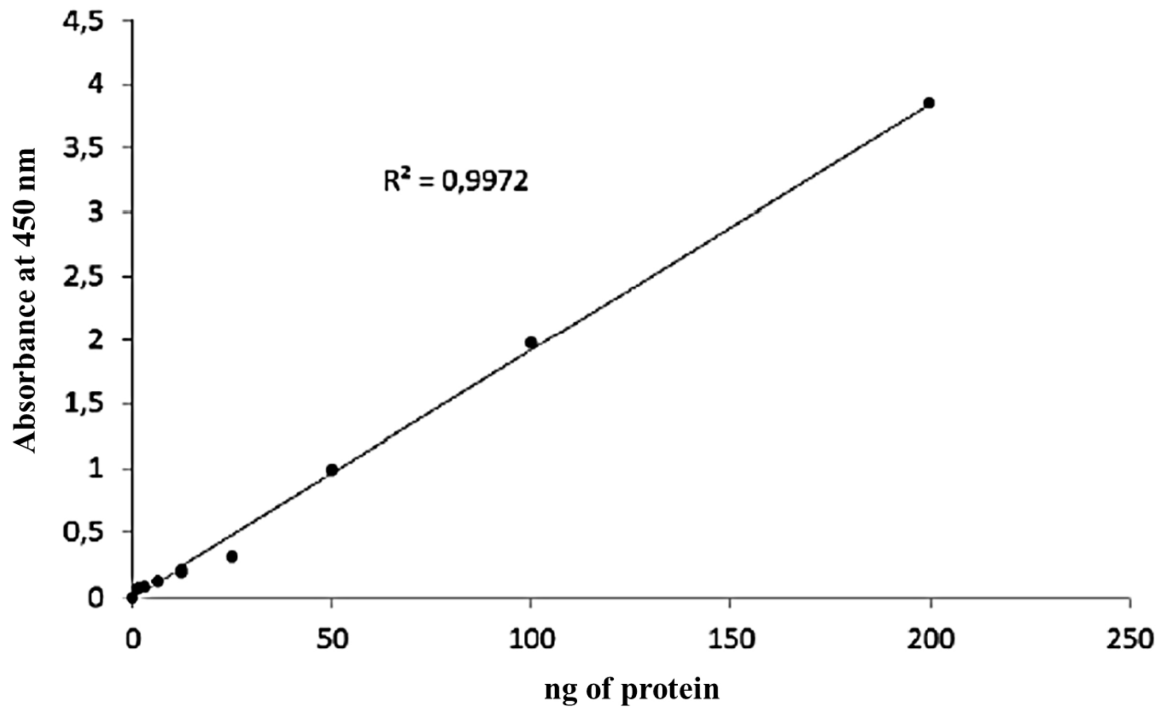

Supplement: Supplementary file 1 [file foods-08-00513-s001.zip › Figure S3.pdf]
